# The regulation of quantitative variation of foliar terpenes in medicinal tea tree *Melaleuca alternifolia*

**DOI:** 10.1186/1753-6561-5-S7-O20

**Published:** 2011-09-13

**Authors:** Hamish Webb, Kulheim Carsten, Lanfear Rob, John Hamill, William Foley

**Affiliations:** 1 Australian National University, Australia; 2 Monash University, Australia

## Background

Terpenes are important mediators of plant-environment interactions as well as often being economically important. Terpenes are biosynthesized via two spatially separate pathways: the MEP pathway in the plastid, the site of monoterpene biosynthesis and the MVA pathway in the cytosol, the site of sesquiterpene biosynthesis (McGarvey and Croteau 1995; Eisenreich *et al*. 1998; Rohmer 1999). Studies using transgenic plants have demonstrated that when the MEP genes *dxs* and *dxr* and MVA pathway gene *hmgr* are up-regulated plants exhibit an increase in foliar terpene concentrations (Chappell *et al*. 1995; Wildung and Croteau 2005; Carretero-Paulet *et al.* 2006). However, as yet, the importance of the expression of these genes has not been demonstrated for naturally occurring quantitative variation in foliar terpene concentrations.

**Figure 1:**
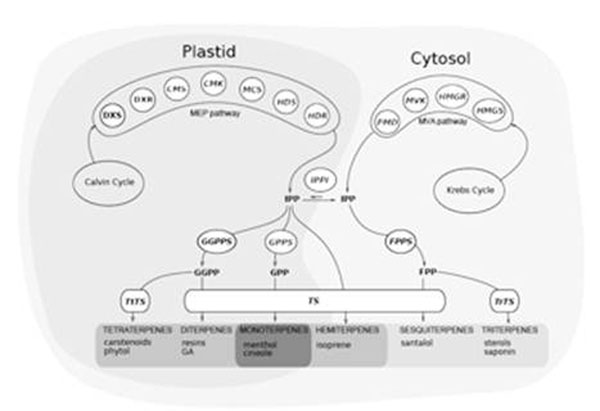
Simplified schematic of the terpene biosynthetic pathway in plants, showing the catalyzing enzymes for each step.

## Aims and methods

*Melaleuca alternifolia*(medicinal tea tree) is a small tree in the family Myrtaceae that is grown in plantations for its valuable terpene-rich leaf oil. We studied the expression of MVA and MEP pathway genes to identify those that are correlated with variation in foliar terpene concentration. Diagnostic primers were designed for seven MEP pathway genes, three MVA pathway genes and three genes downstream of both pathways. Transcript abundance for all 13 genes was then quantified from 48 individuals on the Fluidigm biomark platform.Foliar terpene concentrations were determined by GC of ethanol extracts containing an internal standard.

## Results

**Figure 2:**
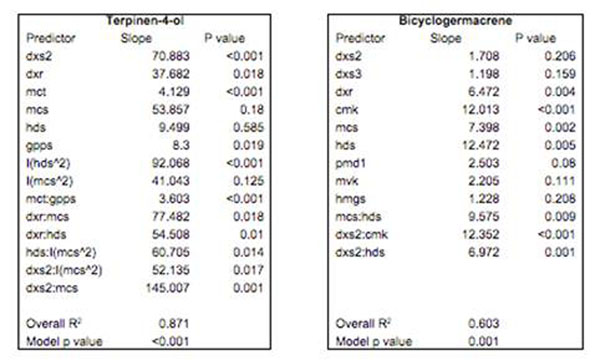
Multiple linear regression models of gene expression against terpinen-4-ol and biocyclogermacrene concentrations

Foliar terpene concentration varied from 5 to 12.2 % of dry matter (DM), terpinen-4-ol concentration from 2.1 to 6.6 % of DM and bicyclogermacrene concentration from 0.1 to 0.5 % of DM. The transcript abundance of five genes within the MEP pathway were correlated significantly with variation in foliar terpinen-4-ol concentrations. The transcript abundance of three MEP pathway genes and one MVA pathway gene were significantly correlated with foliar bicyclogermacrene (the most abundant sesquiterpene) concentrations. However, the expression of the genes within both pathways were co-dependent and showed strong pair-wise correlations within pathways. Therefore, multiple regression models were formulated to account for interactions amongst the genes. A multiple regression model using only the genes in the MEP pathway explained 87% of variation in foliar terpinen-4-ol concentrations (Figure 2). A multiple regression model of the genes within both the MEP and MVA pathways explained 60% of variation in foliar bicyclogermacrene concentrations (Figure 2). Two sub-models using only the genes from either pathway explained significantly less variation in bicylclogermacrene concentrations than the full model (MEP only, *r^2^*= 0.5; MVA only, *r^2^*= 0.15) and indicated that both pathways contribute to the pool of isopenthyl pyrophosphate (IPP) used to biosynthesize bicyclogermacrene.

## Discussion

Several studies have shown that the expression of *dxr* and *dxs* has a strong influence on foliar terpene yield; however, this has only ever been demonstrated in transgenic plants and never on naturally occurring, quantitative variation in terpene concentrations. Our results show that a large proportion of the quantitative variation of foliar terpene concentration in *M. alternifolia* can be explained by quantitative variation of transcript abundance of genes in the early stages of terpenoid biosynthesis. A number of MEP pathway genes are correlated with variation in both the monoterpene terpinen-4-ol as well as the sesquiterpene bicyclogermacrene, which is also correlated with one MVA pathway gene. The multiple regression models for both terpinen-4-ol and bicyclogermacrene, each explained a significant amount of variation in the concentrations of each terpene.

The correlation between monoterpenes and transcripts from the MEP pathway was expected as they both occur in the plastid, however we also found a significant correlation between MEP pathway genes and bicyclogermacrene, a sesquiterpene synthesized in the cytosol. These correlations, as well as the results of the multiple regression analysis, strongly suggests that in *M. alternifolia*,the IPP from the plastid contributes significantly to the pool of IPP used for sesquiterpene biosynthesis.

## Conclusions

This is the first study that shows the importance of variation in transcript abundance of terpene biosynthesis genes in determining naturally occurring variation in foliar terpene concentrations. The results also suggest that IPP originating in the plastid contributes significantly to the pool of precursors used for sesquiterpene biosynthesis in the cytosol in *M. alternifolia*. These results open the way for future studies to identify markers that can be used to improve early selection of plants with an enhanced yield and thus greater profitability.
